# Tension Pneumocephalus Caused by a Communicating Pneumothorax via a Nerve Root Avulsion

**DOI:** 10.7759/cureus.67317

**Published:** 2024-08-20

**Authors:** Benny Brandvold, Donghy Lee, Judner Garcon

**Affiliations:** 1 Neurological Surgery, Benefis Health System, Great Falls, USA; 2 Medical School, Touro College of Osteopathic Medicine, Great Falls, USA

**Keywords:** mount fuji sign, pneumocephalus, thoracic trauma, complications, neurosurgery, tension pneumocephalus

## Abstract

Tension pneumocephalus (TP) is an unusual and potentially life-threatening condition characterized by a large volume of intracranial air causing compression and displacement of the underlying cerebral cortex. The symptoms of TP are non-specific. Diagnosis is generally made by demonstration of the classic “Mount Fuji” sign on computed tomography imaging. TP is most commonly seen in the early postoperative period after craniotomy or trauma involving fractures through the pneumatized sinuses. We present a rare case of TP which resulted from an inadequately decompressed pneumothorax communicating with root avulsions of C8 and T1.

## Introduction

Although pneumocephalus (asymptomatic intracranial air) resulting from trauma or neurosurgery is a common occurrence, its progression to tension pneumocephalus (TP) (symptomatic intracranial air) is a rare phenomenon [1]. The diagnosis is often delayed because the signs and symptoms are non-specific. Headache, deteriorating mental status, seizures, dizziness, confusion, nausea, and focal neurological deficits have been described in association with TP [2]. TP can be diagnosed with a head computed tomography (CT) revealing air in the frontal region shaped like the silhouette of Mount Fuji [3]. Incidental pneumocephalus usually resolves on its own, but TP often requires intervention. There have been a limited number of cases reported involving penetrating thoracic trauma with dural injury resulting in TP. We present the case of a 38-year-old female involved in a motor vehicle accident who developed TP from an inadequately treated pneumothorax which communicated with C8 and T1 nerve root avulsions. Appreciation of the “Mount Fuji” sign on CT imaging allowed for timely diagnosis and management with chest tube decompression, Trendelenburg positioning, and 100% oxygen administration.

## Case presentation

**Figure 1:**
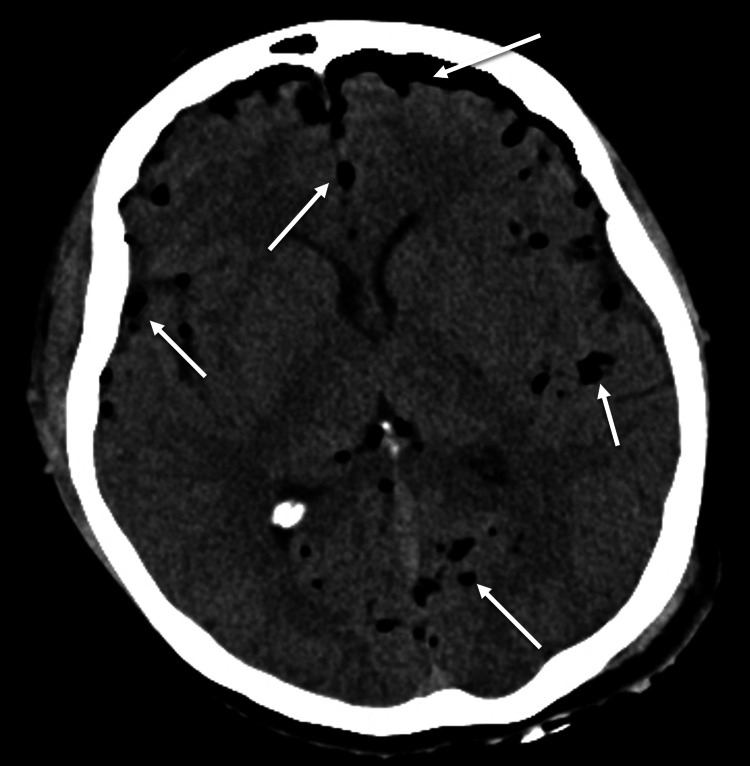
Initial head computed tomography showing pneumocephalus (arrows) without mass effect.

**Figure 2:**
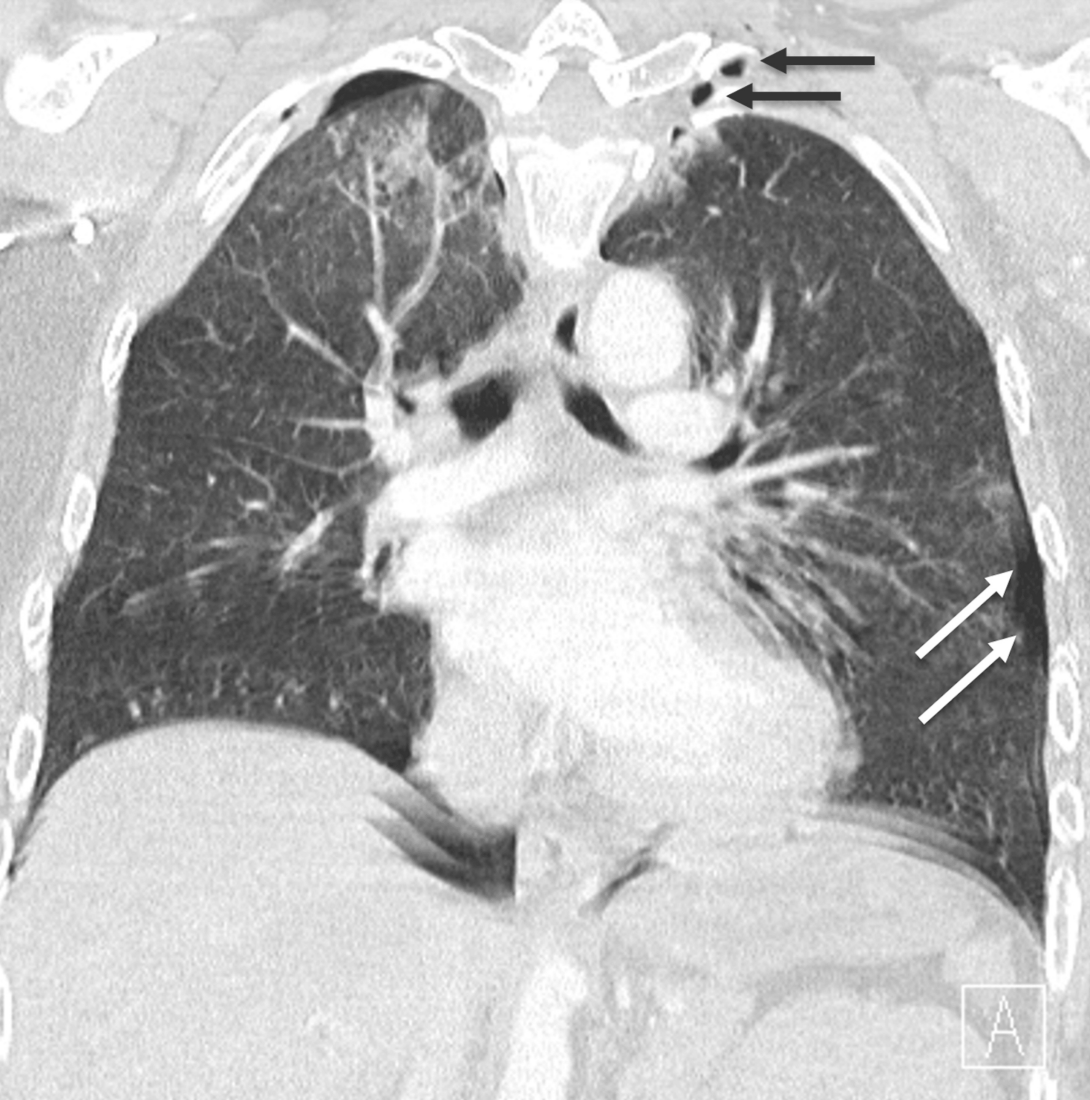
Chest computed tomography showing small left pneumothorax (white arrows) and gas in soft tissues adjacent to lateral masses (gray arrows).

**Figure 3:**
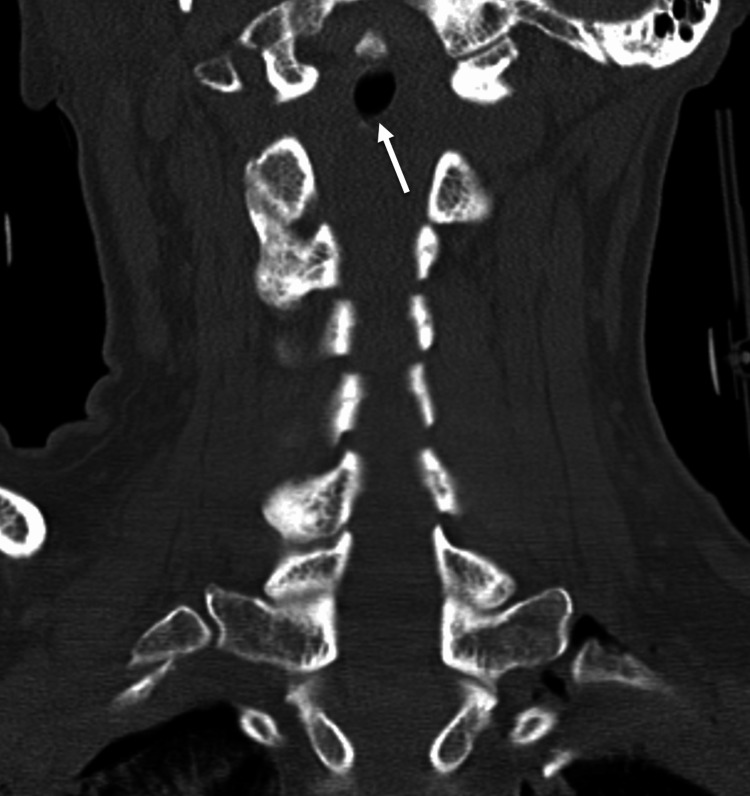
Coronal computed tomography of the cervical spine demonstrating intradural gas (arrow) and gas adjacent to neuroforamina.

A 38-year-old female was admitted to the emergency department after a rollover motor vehicle accident. On arrival at the emergency room, the patient was awake and alert. The patient complained of headache, chest pain, left forearm pain, and numbness. The examination demonstrated intrinsic weakness and hyperpathia in her left hand. Radiographic workup demonstrated moderate pneumocephalus (Figure 1), a small left pneumothorax (Figure 2), minimal intradural air in the cervical and lumbar spine, and gas adjacent to the lateral masses and neuroforamina on chest CT (Figure 3), as well as non-displaced 2-6 rib fractures on the left on X-ray. No skull fractures were visualized on bone windows to explain the pneumocephalus.

**Figure 4:**
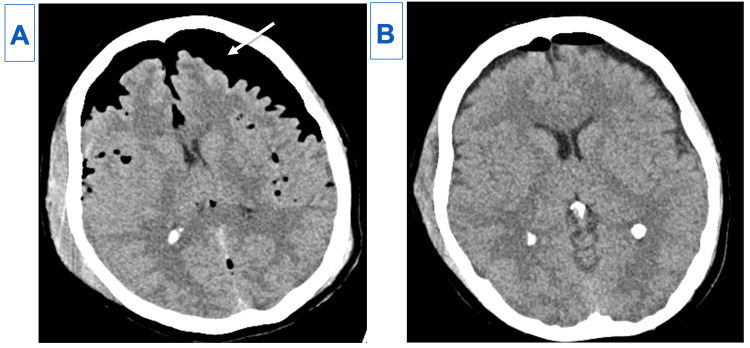
(A) Follow-up computed tomography (CT) the next AM showing tension pneumocephalus with the classic Mount Fuji sign (arrow). (B) 72 hours post-treatment CT showing near resolution of tension pneumocephalus.

**Figure 5:**
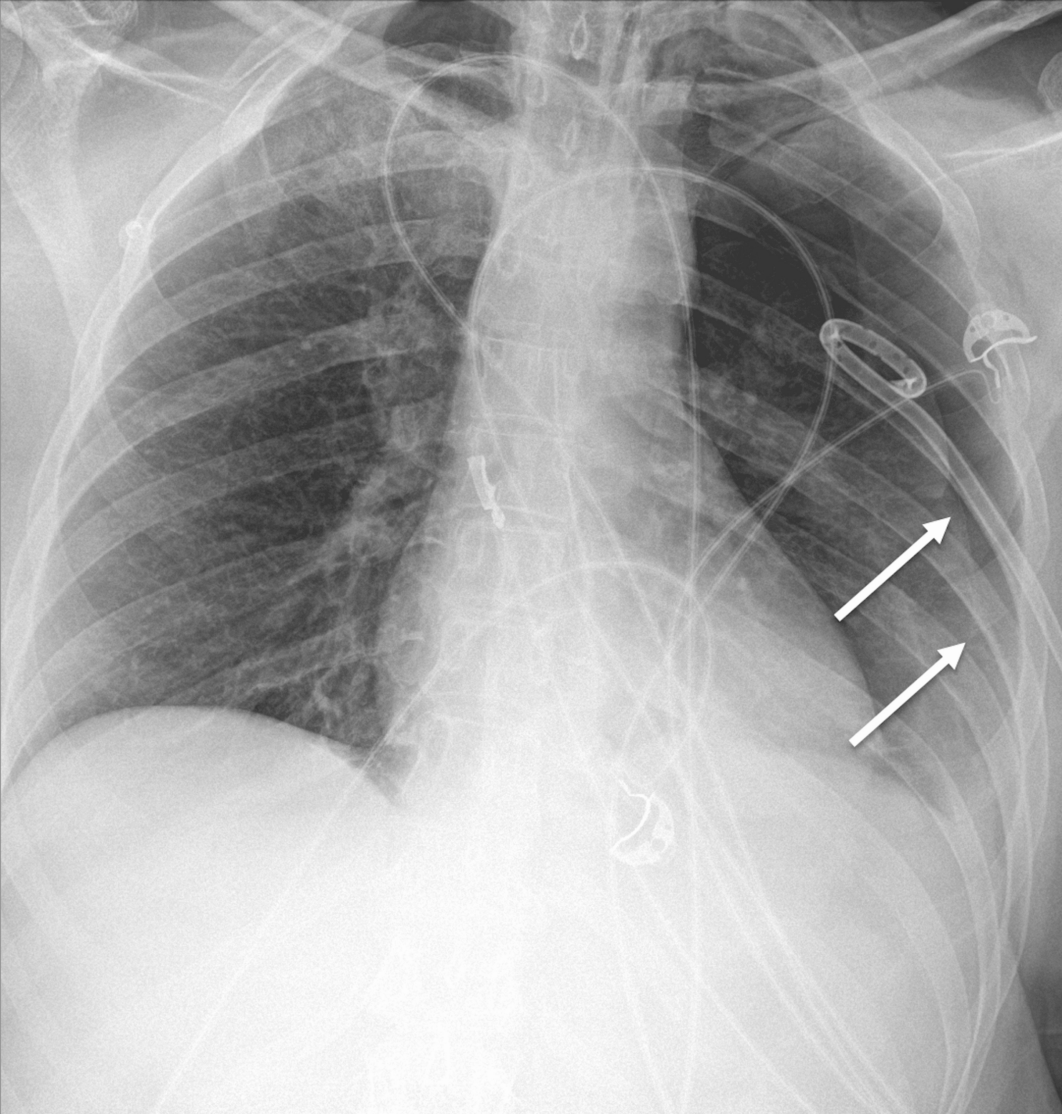
Left pneumothorax (arrows) despite corrective pigtail catheter.

A pigtail thoracostomy was placed and she was admitted to the intensive care unit with the head of the bed elevated. Routine head CT the following morning showed a substantial increase in her pneumocephalus with the classic “Mount Fuji” sign (Figure 4A). Chest X-ray revealed an increase in her pneumothorax. An 8-French chest tube was placed to decompress her pneumothorax (Figure 5). She was placed in the Trendelenburg position with a 100% non-rebreather mask. Follow-up CTs demonstrated a marked decrease in the pneumocephalus over 48 hours (Figure 4B).

**Figure 6:**
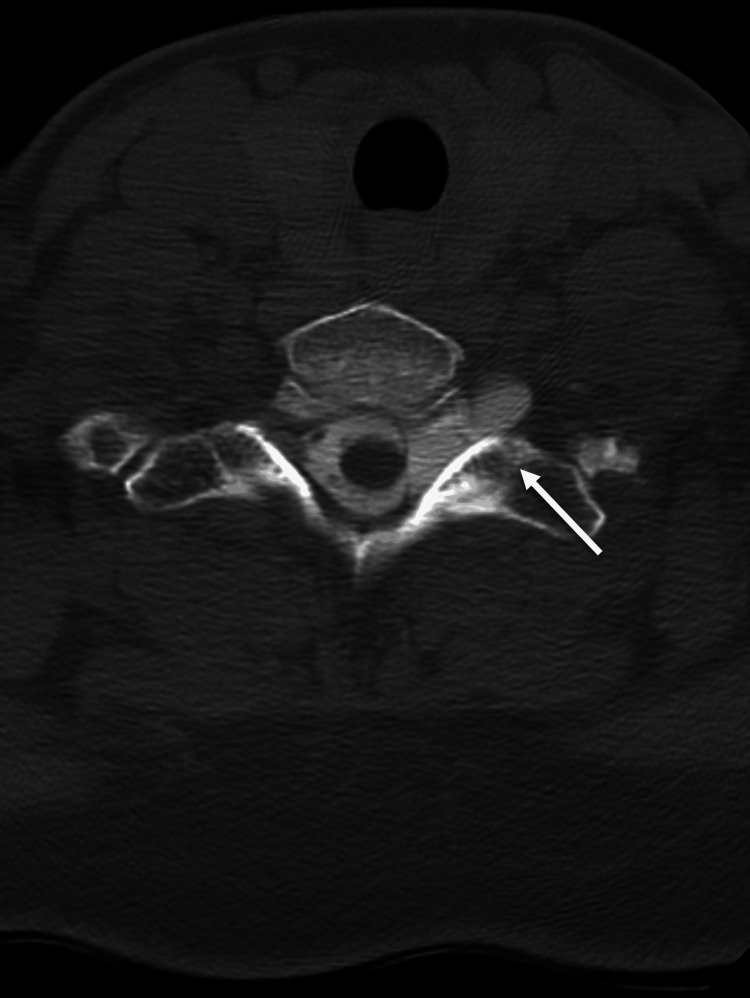
Delayed computed tomography myelogram showing pseudomeningocele typical of root avulsion (arrow).

At the three-month follow-up, the patient exhibited Horner’s syndrome with marked extrinsic weakness and medial hand sensory loss. A myelogram (Figure 6) showed pseudomeningoceles of the C8 and T1 root sleeves suggestive of nerve root avulsions and nerve root sleeve injury.

## Discussion

TP is a potentially life-threatening condition that results when a significant amount of intracranial air causes compression of the underlying cerebral cortex [4]. This condition most commonly occurs after intracranial surgery or trauma involving the pneumatized sinuses at the skull base. Clinical signs and symptoms are non-specific and can include headache, deteriorating mental status, seizures, dizziness, confusion, nausea, and focal neurological deficits [2]. Two theories attempt to explain how TP develops, namely, the “inverted bottle effect” and the “one-way air valve” [3]. The “inverted bottle effect” theorizes that cerebrospinal fluid (CSF) loss may draw in a large volume of air causing the pneumocephalus. Both traumatic or iatrogenic loss of CSF can cause this; therefore, a subtle fracture or CSF leak should be searched for clinically or radiologically when TP is indicated on the CT scan [5]. The “one-way air valve” theory states that a tear in the dura allows air to be forced through a rent in the dura, trapping it within the intracranial space. This is a well-accepted theory in the creation of tension pneumothoraces with coughing, sneezing, and positive pressure ventilation [6].

We present a patient who developed TP (Figure 4A) following a motor vehicle accident, where she sustained a pneumothorax (Figure 5) as well as C8 and T1 nerve root and sleeve avulsions (Figure 6). This created a rent in the dura, allowing gas from her pneumothorax to be forced into the dura and travel to the intracranial space. This became the primary route of dispersion when she was placed in the head of the bed position.

Previous cases reported TP caused by a fistula created by penetrating trauma [4] and through an unvalved ventriculopleural shunt [7]. Unlike these other cases, we were able to avoid cranial surgery. Given the pressure of the gas in the pleural space was greater than the intracranial pressure, this had to be addressed first by decompressing the pneumothorax [3]. The patient was placed in the Trendelenburg position, which allowed some of the intracranial air to dissipate into the spine. The Trendelenburg position also allowed fluid to accumulate at the apex of the pleural space, essentially creating an epidural blood patch sealing the dural rent and closing the fistula. The patient was also placed on a 100% non-rebreather oxygen mask to hasten the removal of intracranial nitrogen by diffusion [3]. Prompt diagnosis of TP and timely treatment with chest tube decompression, Trendelenburg positioning, and 100% oxygen administration allowed the pneumocephalus to gradually resolve (Figure 4B).

## Conclusions

TP is a potentially life-threatening condition that results when a significant amount of intracranial air causes compression of the underlying cerebral cortex. In our case, the pressure gradient caused by the pneumothorax created a one-way valve across a dural rent that allowed air to be trapped in the intradural space. Management of the process required addressing the pneumothorax to alleviate the pressure gradient. By placing the patient in the Trendelenburg position, the air was redistributed into the spine. Further, 100% oxygen administration facilitated the reabsorption of the intradural air by diffusion into the bloodstream.
